# Rapid HILIC-Z ion mobility mass spectrometry (RHIMMS) method for untargeted metabolomics of complex biological samples

**DOI:** 10.1007/s11306-022-01871-1

**Published:** 2022-02-28

**Authors:** Martina Pičmanová, Tessa Moses, Joan Cortada-Garcia, Georgina Barrett, Hannah Florance, Sufyan Pandor, Karl Burgess

**Affiliations:** 1 Institute of Quantitative Biology, Biochemistry and Biotechnology, University of Edinburgh, Max Born Crescent, Edinburgh, EH9 3BF UK; 2 EdinOmics, University of Edinburgh, Max Born Crescent, Edinburgh, EH9 3BF UK; 3 Agilent Technologies UK Limited, Cheadle Royal Business Park Stockport, Cheshire, SK8 3GR UK

**Keywords:** Untargeted metabolomics, HILIC, Ion mobility, High throughput

## Abstract

**Introduction:**

Recent advances in high-throughput methodologies in the ‘omics’ and synthetic biology fields call for rapid and sensitive workflows in the metabolic phenotyping of complex biological samples.

**Objective:**

The objective of this research was to evaluate a straightforward to implement LC–MS metabolomics method using a commercially available chromatography column that provides increased throughput. Reducing run time can potentially impact chromatography and therefore the effects of ion mobility spectrometry to expand peak capacity were also evaluated. Additional confidence provided via collision cross section measurements for detected features was also explored.

**Methods:**

A rapid untargeted metabolomics workflow was developed with broad metabolome coverage, combining zwitterionic-phase hydrophilic interaction chromatography (HILIC-Z) with drift tube ion mobility-quadrupole time-of-flight (DTIM-qTOF) mass spectrometry. The analytical performance of our method was explored using extracts from complex biological samples, including a reproducibility study on chicken serum and a simple comparative study on a bacterial metabolome.

**Results:**

The method is acronymised RHIMMS for rapid HILIC-Z ion mobility mass spectrometry. We present the RHIMMS workflow starting with data acquisition, followed by data processing and analysis. RHIMMS demonstrates improved chromatographic separation for a selection of metabolites with wide physicochemical properties while maintaining reproducibility at better than 20% over 200 injections at 3.5 min per sample for the selected metabolites, and a mean of 13.9% for the top 50 metabolites by intensity. Additionally, the combination of rapid chromatographic separation with ion mobility allows improved annotation and the ability to distinguish isobaric compounds.

**Conclusion:**

Our results demonstrate RHIMMS to be a rapid, reproducible, sensitive and high-resolution analytical platform that is highly applicable to the untargeted metabolomics analysis of complex samples.

**Supplementary Information:**

The online version contains supplementary material available at 10.1007/s11306-022-01871-1.

## Introduction

In tandem with the well-established ‘omics’ approaches of genomics, transcriptomics and proteomics, the burgeoning field of metabolomics adds a further dimension to our understanding of the biology of organisms and their response to environmental stimuli, metabolic engineering and synthetic biology interventions. With the rapid development of high-throughput technologies, powerful data-processing software packages and sophisticated statistical tools, the potential of metabolomics to provide deeper insight into cellular physiology and metabolic networks is ever expanding. Liquid chromatography–mass spectrometry (LC–MS) is currently the most widely used analytical platform for untargeted metabolic phenotyping, due to its versatility in metabolite coverage and the sensitivity of the instrumentation (Gertsman & Barshop, 2018; Gika et al., 2019). Depending on the specific choice of chromatographic method and type of mass spectrometer, many hundreds to thousands of metabolic features with diverse chemical properties can be identified and semi-quantified, using an untargeted approach.

However, to explore complex biological systems requires more powerful analytical methods, allowing the efficient separation of the different constituents, increased resolution, and higher confidence in metabolite annotation and identification (D’Atri et al., 2018; Dodds & Baker, 2019; Haggarty & Burgess, 2017; Mairinger et al., 2018). Incorporating ion mobility spectrometry (IMS) into LC–MS-based metabolomics methodologies enables structural evaluation of small molecules in complex matrices by assessing the drift times (tD) and collision cross section (CCS) values for individual features (via IMS), in addition to chromatographic retention times (via LC) and *m/z* values (via MS); these parameters combined provide increased resolution and more accurate identification of small molecules present in complex biological and environmental samples (Gabelica & Marklund, 2018; Gika et al., 2019; Odenkirk & Baker, 2020).

Multidimensional LC-IM-MS-based untargeted metabolomics has been demonstrated to be a useful tool across the breadth of metabolomics applications, and has found application in clinical research for the detection of novel biomarkers, in the assessment/authentication of herbal formulations and beverages, for discrimination between crop varieties and in other biological studies (Avula et al., 2020; Causon et al., 2019; Claassen et al., 2019; Jia et al., 2019; Lacalle-Bergeron et al., 2020; Montero et al., 2020; Yang et al., 2018). The method commonly employed in these applications is RP/HILIC UPLC-TWIMS/DTIMS-qTOF (reverse phase or hydrophilic interaction ultra-performance liquid chromatography-traveling wave or drift tube ion mobility-quadrupole time of flight mass spectrometry), but less common combinations, such as HILIC-FAIMS-TOF (miniaturised high-field asymmetric waveform ion mobility spectrometry-TOF), CZE-DTIMS-TOF (capillary zone electrophoresis-DTIMS-TOF), chromatography-free MALDI-TIMS-TOF imaging mass spectrometry (matrix-assisted laser desorption/ionisation-trapped ion mobility spectrometry-TOF) or LAESI-TWIMS-qTOF (laser ablation electrospray ionization-TWIMS-qTOF), have also been exploited in metabolomics studies (Drouin et al., 2021; Neumann et al., 2020; Stopka & Vertes, 2020; Szykula et al., 2019).

The addition of IMS in the metabolomics workflow provides an attractive possibility for the development of rapid methods without compromising too much on the coverage of metabolites. However, a certain degree of reduction in peak capacity and feature detection is inevitable in such express chromatographic methods. Typically, the time scale of data acquisition in LC-IM-MS experiments is in the minute range for the chromatographic step, in the millisecond range for IMS and in the microsecond range for MS (D’Atri et al., 2018). The choice of appropriate chromatographic column and elution gradient can significantly decrease the time required for the chromatography from tens of minutes to less than four minutes per sample, while still allowing many thousands of molecular features to be detected (King et al., 2019; Rainville et al., 2017).

Here, we present a method acronymised as RHIMMS (rapid HILIC-Z Ion mobility mass spectrometry), uniquely combining zwitterionic-phase hydrophilic interaction chromatography (HILIC-Z) with drift tube ion mobility-quadrupole time-of-flight (DTIM-qTOF) mass spectrometry for rapid untargeted metabolomics analysis of biological samples. RHIMMS enables swift data acquisition, both in negative (high pH) and positive (low pH) ionization modes for the detection of a wide range of metabolites; it generates thousands of molecular features and allows metabolite identification with an increased degree of confidence, based on accurate mass, drift time and collision cross section values for individual features. The sensitivity and reproducibility of the method were explored using the metabolome of chicken serum and the method’s performance was evaluated using a bacterial metabolome by interrogating both intra- and extracellular metabolites.

## Materials and methods

### Chemicals

Ammonia solution 0.88 SG, formic acid, and Optima™ LC/MS grade water, acetonitrile and methanol were purchased from Fisher Scientific (Loughborough, UK). Ammonium acetate was from Sigma-Aldrich (Gillingham, UK). Chloroform, ammonium formate and hydrogen peroxide (30 wt%, non-stabilized) were obtained from Thermo Fisher Acros Organics (Geel, Belgium). Amino acid standards mix was from Merck (Gillingham, UK), maltose monohydrate and sucrose were from VWR (Lutterworth, UK). The ESI-L low concentration tuning mix and ES-TOF reference mass solution kit were purchased from Agilent Technologies (Santa Clara, CA).

### Biological sample preparation

Chicken serum, New Zealand origin (Gibco 16110-082) was extracted in a 1:10 dilution with ice-cold chloroform/methanol/water (1:3:1). The extract was centrifuged at 13,000×*g* for 10 min before RHIMMS analysis. To prepare bacterial metabolomes, overnight pre-culture of *Rhizobium leguminosarum* bv. *trifolii* (3 colonies pooled in each replicate) was used to inoculate 30 mL Luria Bertani (LB) broth to OD_600_ of 0.1, followed by the addition of hydrogen peroxide to a final concentration of 2 mM. The hydrogen peroxide-treated and untreated cultures were incubated at 30 °C, 160 rpm for 6 h (exponential growth phase) and OD_600_ was measured at the end of incubation for ex post normalization. 1.9 mL cultures were quenched rapidly by immersion of tubes in a dry ice/ethanol bath for 10 s while being shaken manually. The quenched samples were centrifuged at 4 °C for 10 min at 1000×*g*, and 1 mL of the supernatant was transferred to a fresh tube for the analysis of spent media. The cell pellets were centrifuged again for 10 min at 2500×*g* and all remaining medium discarded. The pellets were suspended in 400 µL of ice-cold chloroform/methanol/water (1:3:1) mixture and incubated in a thermomixer (Eppendorf Thermomixer R, Hamburg, Germany), for 1 h at 4 °C and 1200 rpm. Lysed cells were centrifuged for 3 min at 13,000×*g* at 4 °C and the supernatants (350 µL) transferred to fresh tubes and stored at − 80 °C until analysis by RHIMMS. For the analysis of spent media, the supernatant recovered from each culture was centrifuged for 3 min at 13,000×*g* at 4 °C, followed by dilution of 10 µL supernatant in 390 µL ice-cold chloroform/methanol/water (1:3:1) and incubation for 5 min at 1200 rpm at 4 °C. Samples were centrifuged for 3 min at 13,000×*g* at 4 °C and supernatants (350 μL) transferred into clean tubes and stored at − 80 °C. Fresh medium (with and without hydrogen peroxide) was processed in the same way as spent medium.

### HILIC chromatography and ion-mobility qTOF mass spectrometry method

Chromatographic separation was performed using either an InfinityLab Poroshell 120 HILIC-Z, 2.1 mm × 50 mm, 2.7 μm column (Agilent Technologies 689775-924, Santa Clara, CA) coupled to InfinityLab Poroshell 120 HILIC-Z, 3.0 mm × 2.7 µm UHPLC guard column (Agilent Technologies 823750-948, Santa Clara, CA), or a SeQuant ZIC-pHILIC, 5 μm polymeric 4.6 mm × 150 mm column (Merck KGaA 1.50461.0001, Darmstadt, Germany) coupled to a SeQuant ZIC-pHILIC Guard 20 × 2.1 mm column (Merck KGaA 1.50437.0001, Darmstadt, Germany). Two different solvent systems of low and high pH were used to run 3.5 min or 30 min gradients on the 50 mm or 150 mm columns, respectively (Table 1). The LC-IM-MS instrumentation consisted of an Agilent 1290 Infinity II series UHPLC system coupled to an Agilent 6560 IM-qTOF (both Agilent Technologies, Santa Clara, CA) with a Dual Agilent Jet Stream Electron Ionization source. Table 1 summarizes the optimized chromatographic and IM-MS conditions. The same general parameters were used for the qTOF only method, with an acquisition rate of 3 spectra/s and 20 V collision energy. A more comprehensive list of IM-qTOF parameters can be found in Supporting Information (Table S1).

**Table 1:** Optimized UHPLC and IM-qTOF parameters

Ultra-performance liquid chromatography (Agilent 1290 infinity II)				
Column type	Poroshell 120 HILIC-Z (PEEK-coated)		SeQuant ZIC-pHILIC (PEEK-coated)	
Parameters	2.1 mm × 50 mm, 2.7 μm		4.6 mm × 150 mm, 5 µm	
Mobile phases	Positive ion mode	Negative ion mode	Positive ion mode	Negative ion mode
A	10 mM ammonium formate in water with 0.1% formic acid, pH 3	10 mM ammonium acetate in water, pH 9	10 mM ammonium formate in water with 0.1% formic acid, pH 3	10 mM ammonium acetate in water, pH 9
B	10 mM ammonium formate in water/ACN (1:9) with 0.1% formic acid, pH 3	10 mM ammonium acetate in water/ACN (1:9), pH 9	10 mM ammonium formate in water/ACN (1:9) with 0.1% formic acid, pH 3	10 mM ammonium acetate in water/ACN (1:9), pH 9
Gradient	Time (min)	% B	Time (min)	% B
	0.00	93	0	80
	1.80	80	15	20
	2.00	70	16	5
	2.30	70	20	5
	2.35	93	21	80
	3.50	93	30	80
Flow rate	0.8 mL/min		0.3 mL/min	
Column temp	30 °C		30 °C	
Injection vol	1 μL		5 μL	

IM-qTOF (Agilent 6560)				
Parameter		Parameter	Positive ion mode (V)	Negative ion mode (V)
Mass range	50–1700 m*/z*	Drift tube entrance volt.	1250	− 1175
IM transient rate	16 (IM transients/frame)	High pressure funnel RF	120	− 100
Max drift time	60 ms	Trap funnel RF	180	− 80
Trap fill time	2500 µs	Rear funnel RF	130	− 90
Trap release time	500 µs	Trap exit grid 1	88.5	− 86
Pulsing sequence length	4-bit	Trap exit grid 2	87	− 84.9

See Table S1 for a comprehensive list of IM-qTOF parameters

### Data acquisition, processing and statistical analysis

Data acquisition and processing were performed using the Agilent MassHunter software suite. Briefly, ion multiplexed data files and calibration files acquired using MassHunter Data Acquisition 10.0 were demultiplexed using the PNNL PreProcessor v2020.03.23 (the default settings for demultiplexing, moving average smoothing, saturation repair and spike removal were applied to the data). The data files were recalibrated for accurate mass and drift time using the AgtTofReprocessUi and the IM-MS Browser 10.0, respectively. Molecular features were extracted in Mass Profiler 10.0 with a retention time tolerance of ± 0.3 min, drift time tolerance of ± 1.5% and accurate mass tolerance of ± (5 ppm + 2 mDa). The raw multiplexed data, the reconstructed demultiplexed data and Mass Profiler feature lists (.cef files) were then utilized in the High Resolution Demultiplexer (HRdm) 1.0 beta v41 for further peak deconvolution (May et al., 2020). Features were re-extracted from HRdm files using Mass Profiler 10.0 and annotated based on accurate mass and CCS values using the Unified CCS Compendium PCDL (Picache et al., 2019) (version 20191101) with CCS value tolerance of ± 1%. Extracted ion chromatograms (EICs) were analyzed in Mass Hunter Qualitative Analysis 10.0 or in Skyline v20.2 Targeted Mass Spec Environment (Adams et al., 2020); the latter was also used for integration of peak areas in the chicken serum extract. Multivariate statistical analysis and pathway enrichment analysis were performed using the MetaboAnalyst 5.0 web-based platform (Chong et al., 2019). The input data were log-transformed and auto-scaled. For *Rhizobium leguminosarum* bv. *trifolii* metabolomics analysis, the list of annotated compounds with their relative intensities was submitted to the Pathway Analysis tool, sample-normalized by OD_600_, log-transformed, auto-scaled and examined against the *Mesorhizobium japonicum* MAFF 303,099 KEGG pathway library, using global test and relative betweenness centrality methods for enrichment analysis and topological analysis, respectively.

## Results and discussion

**Fig. 1:**
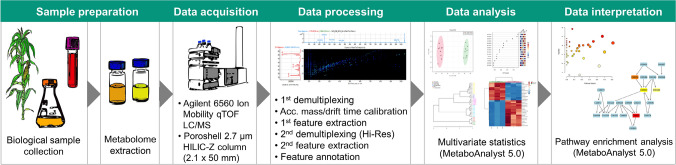
Workflow of untargeted metabolomics approach using RHIMMS method

RHIMMS metabolomics analysis follows the typical workflow of metabolomics studies, starting with biological sample preparation (collection and metabolite extraction), continuing with data acquisition, data processing, statistical analysis, and culminating in data interpretation (Fig. 1). Any type of biological material extracted with a suitable method can be used for the analysis. The rapid 3.5-min LC-IM-MS data acquisition time (both in positive and negative ionization modes) enables the expeditious analysis of a large number of samples, which in turn makes the rapid phenotyping of hundreds of engineered organisms, combinatorial libraries and environmental samples feasible. The bottleneck in the workflow is then determined by limitations in the computational power, and the size and complexity of the dataset used for data processing. The RHIMMS data processing procedure consists of six steps, employing a suite of five different programs, enabling high-resolution peak deconvolution as described in the Experimental Section. A list of molecular features and their annotations, based on comparison of accurate masses and CCS values against selected personal compound databases and libraries (PCDLs), is then utilized for the statistical analysis and interpretation of the data in a biological context. Once processed, the data is in a spreadsheet format which can be analyzed by any downstream statistical package, such as MetaboAnalyst (Chong et al., 2019).

### RHIMMS performance characteristics

**Fig. 2:**
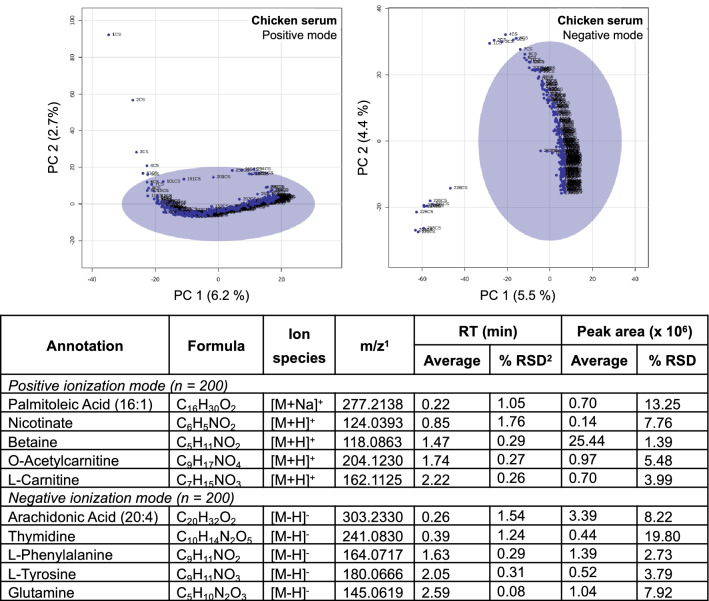
Intra-batch data consistency and reproducibility based on multiple injections of chicken serum extract using RHIMMS. The PCA score plots show negligible variation over the vast majority of injections. Injections 1–5 in positive ionization mode (n = 300; 8881 molecular features), and injections 1–7 and 218–230 in negative ionization mode (n = 230; 3422 molecular features) fall outside the 95% confidence region of the cluster. List of ten selected annotations in chicken serum extract and the deviation of their retention time (RT) and peak area across 200 consecutive injections (recommended batch size) in positive and negative ionization modes are tabulated. ^1^Measured m/z, ^2^*RSD* relative standard deviation

A chicken serum metabolome extract was selected to perform 300 continuous injections in positive ionization mode and 230 continuous injections in negative ionization mode, to assess the requirement for instrument equilibration or ‘burn in’ and the duration of stability after equilibration was achieved (Dunn et al., 2011). Principal component analysis (PCA) score plots, generated using the 8881 and 3422 molecular features detected in positive and negative ionization modes, respectively, demonstrate good reproducibility (< 20% RSD) over the majority of injections (Fig. 2, Table S2). Features vary during injections one to five in positive mode and one to seven in negative mode, and therefore a minimum of five stabilization injections in positive mode and seven injections in negative mode are recommended for system equilibration. As good scientific practice and in order to standardize the injection regime across the two ionization modes, we suggest using eight equilibration samples at the beginning of any RHIMMS experiment. While there is no significant deviation in the sample clustering at the end of the 300 runs in positive ionization mode, 12 injections at the tail end in negative ionization mode fall outside the 95% confidence region of the cluster. Therefore when acquiring RHIMMS data in negative ionization mode, caution is recommended in running a batch sized beyond 200 samples. Once again, as good practice and to standardize the workflow across the two ionization modes, we suggest limiting RHIMMS batches to a maximum of 200 samples. This observation compares favorably with Dunn et al., where 120 and 100 runs were recommended as a maximum for reversed phase LC–MS and gas chromatography GC–MS, respectively (Dunn et al., 2011).

Whereas the intra-batch effect over a large number of consecutive injections was minimal, an inter-batch effect was observed for two batches of chicken serum extract (100 injections each) in both positive and negative ionization mode (Fig. S1). Such variation, caused by system cleaning, calibration, small changes in mobile phase composition, and MS detection sensitivity/resolution, are to be expected and may be ameliorated by applying batch correction methods (Liu et al., 2020; Sanchez-Illana et al., 2018).

To assess the reproducibility of the RHIMMS method during maximal recommended batch sizes (200 samples, as mentioned above), the batch of chicken serum described in Fig. 2 was evaluated by calculating the relative standard deviation (RSD, expressed as a percentage) of retention time and peak area, for five metabolites annotated based on *m/z* and CCS value match against the Unified CCS Compendium PCDL in each ionization mode (Table in Fig. 2). The retention times and peak areas were measured and averaged across 200 consecutive injections of chicken serum in positive and negative mode (the first eight equilibration injections and injections 209 to 300 in positive mode and 209–230 in negative mode were removed from the datasets). RHIMMS showed good reproducibility, both in peak retention, with RSD values below 1.8%, and in peak area, with RSD values ranging from 1.39 to 19.8% (Table in Fig. 2). Restricting the batch size to remove the burn in samples and samples post-208 has improved the RSD values for both peak retention and signal intensity, however thymidine still shows high variability (19.8% RSD for peak area) due to outliers in the 200 samples analysed. These outliers appear to have resulted from feature extraction in our data processing workflow. Very similar % RSD values were reported by King et al. for selected polar metabolites across 17 QC injections (pooled rat urine), using BEH amide HILIC-TWIM-qTOF method in positive ionization mode with 3.3 min acquisition time (King et al., 2019).

### Comparison of RHIMMS with conventional methods

To evaluate its performance further, RHIMMS was compared with conventional 30-min long methods used in our laboratory (Creek et al., 2011). While comparison to the larger format ZIC-pHILIC system may appear incongruous, zwitterionic stationary phase columns remain unusual as applied to metabolomics and the ZIC-HILIC stationary phase represents the most commonly used of these solutions. The choice of the polymer-based ZIC-pHILIC allows high-pH buffers to be used, providing improved separation and ionisation of organic acids and phosphates—the primary constituents of central metabolism, and is matched by the pH tolerance of the HILIC-Z system.

Therefore, a chicken serum extract was analyzed with RHIMMS or conventional methods based on the ZIC-pHILIC (Merck Sequant) column and IM-qTOF (30-min IM-qTOF) (i), or qTOF (30-min qTOF) (ii), only mass spectrometry. These comparisons allow relative assessment of the performance of the stationary phase composition and overall metabolomics performance between ZIC-pHILIC and HILIC-Z (i), as well as the impact on general metabolomics performance of the ion mobility feature (ii).

In both ionization modes combined, comparable numbers of molecular features were extracted (2563 and 2613 for RHIMMS and 30-min IM-qTOF, respectively) and annotated (139 and 159 for RHIMMS and 30-min IM-qTOF, respectively, based on *m/z* and CCS matches against the Unified CCS Compendium PCDL) (Picache et al., 2019). A smaller number of features (1366) and larger number of annotations (275, based on *m/z* only using the same CCS library; including multiple IDs and dubious annotations) were obtained from qTOF alone.

**Fig. 3:**
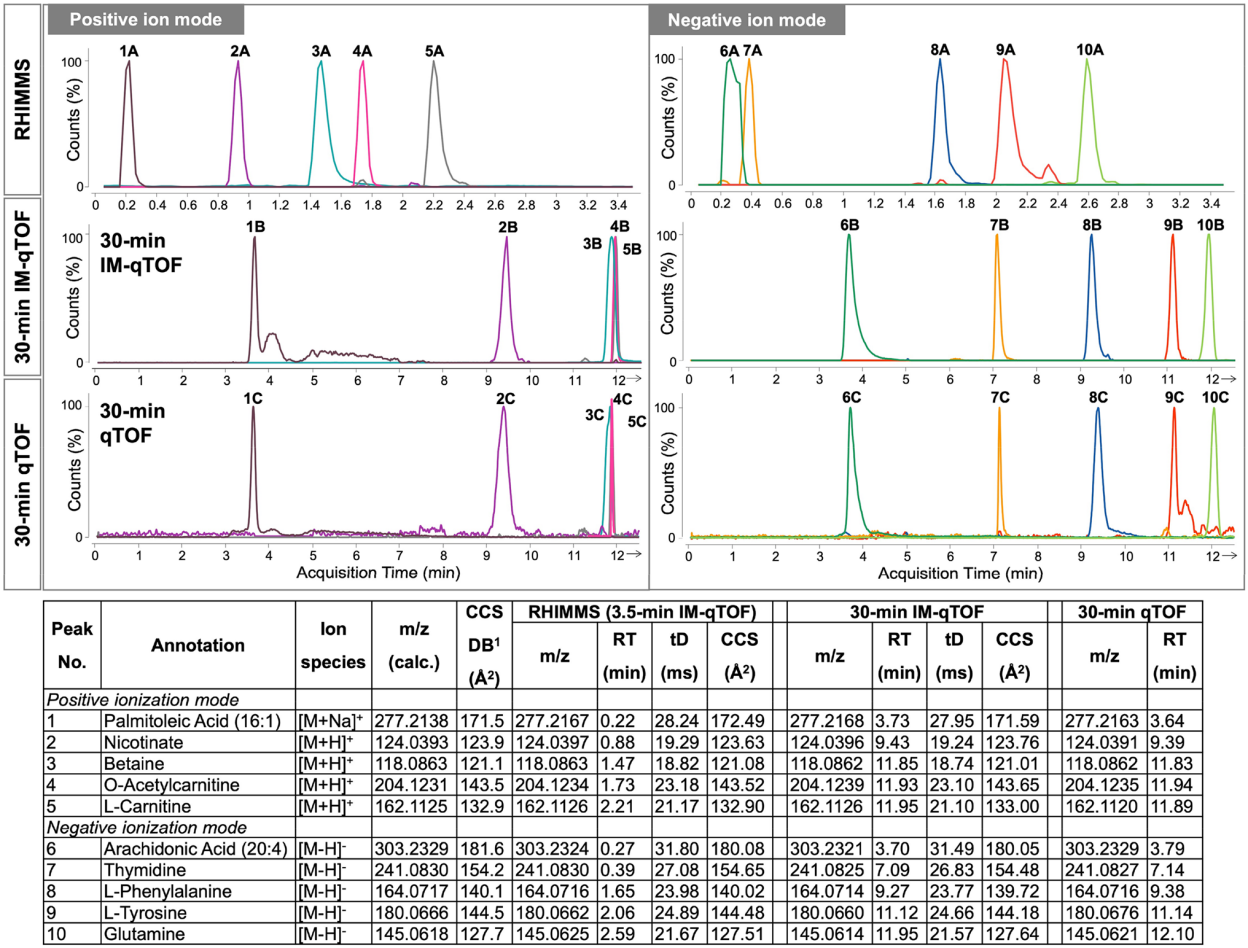
Comparison of extracted ion chromatograms (EICs) obtained from RHIMMS (3.5-min HILIC-Z-IM-qTOF; (A), 30-min ZIC-pHILIC-IM-qTOF (B) and 30-min ZIC-pHILIC-qTOF (C), analyses in positive and negative ionization modes. EICs show selected metabolites from chicken serum extract, putatively identified as (1) Palmitoleic acid (16:1), (2) Nicotinate, (3) Betaine, (4) *O*-Acetylcarnitine, (5) l-Carnitine, (6) Arachidonic acid, (7) Thymidine, (8) l-Phenylalanine, (9) l-Tyrosine, (10) Glutamine. EICs are scaled to the largest in each chromatogram. Measured m/z, retention time (RT), drift time (tD) and CCS values for annotated metabolites, as obtained from each method are tabulated for comparison. ^1^Experimentally derived CCS values (DB) from Unified CCS Compendium

Ten metabolites (presented in Fig. 2) were then subjected to detailed comparison. The overlayed extracted ion chromatograms (EICs) of the ten annotated metabolites, as obtained from the three methods, are shown in Fig. 3 (peaks one to five in positive mode, peaks six to ten in negative mode). The ten selected metabolites have different chemical properties (i.e. the positive mode compounds comprise a non-polar fatty acid, organic acid, amine, quaternary amine and acetylated amine, the negative mode compounds contain a non-polar fatty acid, a deoxyribonucleoside and three amino acids with varying properties) and their peaks exhibit a wide dynamic range, with a difference of up to two orders of magnitude in peak intensity, demonstrating the high sensitivity of the analytical system. For the sake of visibility, the EICs presented are scaled to the largest in each chromatogram. The peaks eluted consistently in the same order across the three methods, with different relative retentions between RHIMMS and our conventional methods. In positive mode, improved separation was achieved by the 3.5-min HILIC-Z method (RHIMMS), whilst co-elution of betaine (*m/z* = 118.0863, peak 3), *O*-acetylcarnitine (*m/z* = 204.1231, peak 4) and L-carnitine (*m/z* = 162.1125, peak 5) was observed when a 30-min ZIC-pHILIC gradient was used (Fig. 3). Except for arachidonic acid (*m/z* = 303.2329, peak 6) and thymidine (*m/z* = 241.083, peak 7), which were not resolved completely in negative mode using RHIMMS, the overall chromatographic performance of RHIMMS was comparable to, or better than that of our conventional methods.

The *m/z* values, retention time (RT), drift time (tD) and CCS values measured for the ten annotated metabolites were further compared between RHIMMS and 30-min IM-qTOF, and the comparison complemented by the *m/z* values and RT obtained from 30-min qTOF method (Table in Fig. 3). In all three methods the *m/z* values fall within the mass tolerance of ± (5 ppm + 2 mDa) and the deviation in the retention time for the two 30-min methods is negligible. The difference in the measured tD values between RHIMMS and 30-min IM-qTOF is ≤ 1%, supporting the veracity of the annotations.

The CCS values represent an important criterion for increased-confidence annotations of the extracted features, based on comparison against experimentally acquired or predicted CCS values in databases/libraries (Zhou et al., 2016, 2020). CCS values are highly specific, universal molecular descriptors, closely related to the molecule’s three-dimensional structure (Odenkirk & Baker, 2020; Paglia et al., 2021). Drift time ion mobility (DTIM), employed in this study, is the only IM technique that provides direct calculations of CCS values from an ion’s drift time (Odenkirk & Baker, 2020). The measured CCS values from both RHIMMS and the 30-min IM-qTOF presented in Fig. 3 differ from the Unified CCS Compendium PCDL values by less than 1%. Thus, the CCS values acquired from the DTIM experiment represent an additional factor that can be utilized for the annotation of metabolites in complex mixtures, in the untargeted metabolomics approach presented here.

**Fig. 4:**
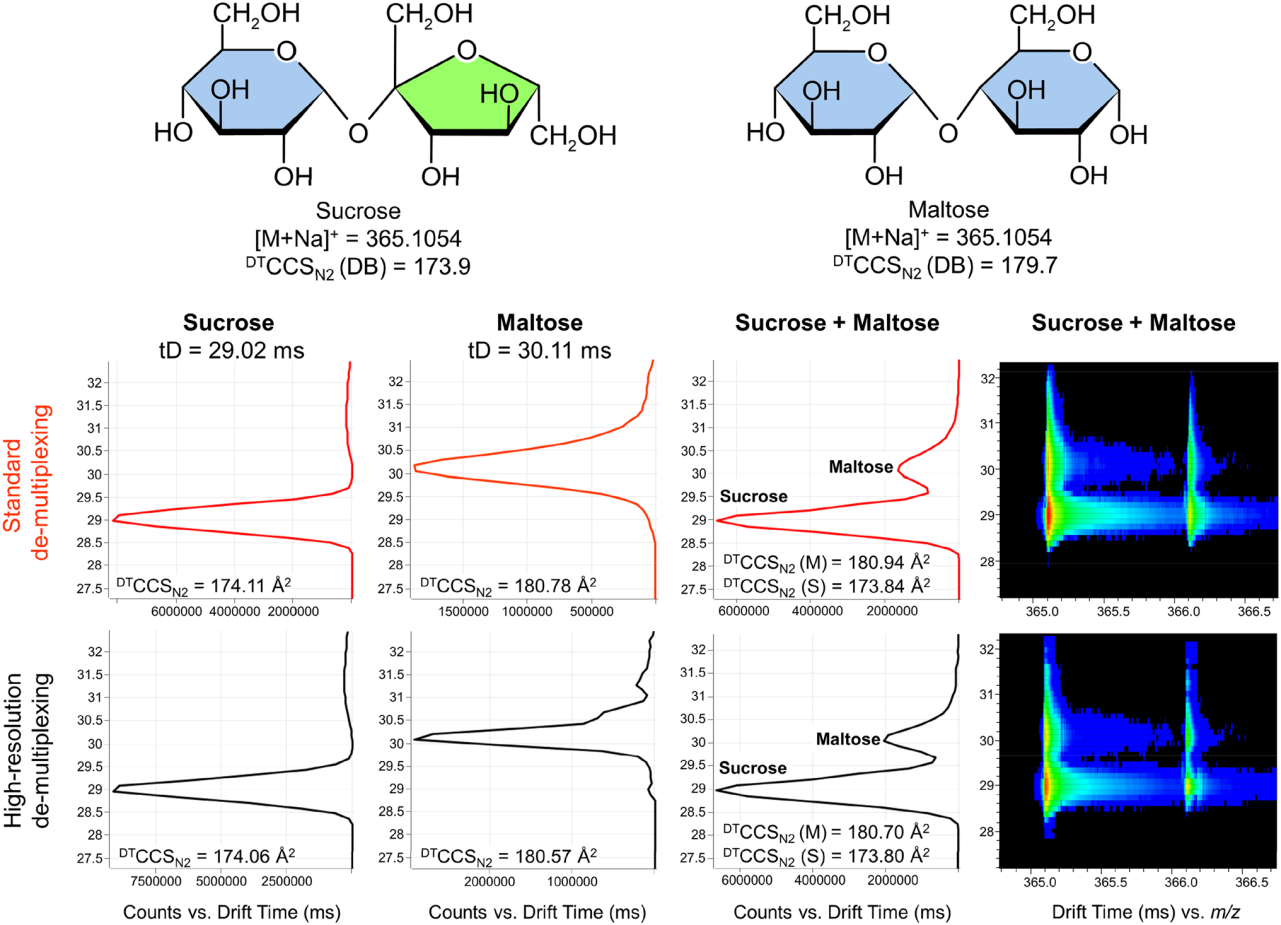
Identification of isomers using RHIMMS ion mobility parameters, as demonstrated for the disaccharides sucrose and maltose, analyzed separately and in a mixture. IMS spectra for [M + Na]^+^ adducts of the disaccharides after standard and high-resolution demultiplexing are presented for comparison. The differentiation between sucrose and maltose is based on the recorded drift time (tD), complemented by the comparison of the measured CCS values with the reference (DB) CCS values (Unified CCS Compendium PCDL)

Besides the advantage of high sensitivity, DTIM-MS separates analytes by their shape and mass relative to their charge, based on gas-phase CCS, and enables separation of structural isomers that are not distinguishable with conventional MS measurements (Dodds et al., 2017; Odenkirk & Baker, 2020). To illustrate the capability of RHIMMS to separate isomers using IM-MS parameters, the co-eluting disaccharides sucrose and maltose ([M + Na]^+^  = 365.1054) were analysed separately and in a mixture (both 1 mg/mL; Fig. 4). The dT of sucrose and maltose differ by 1.09 ms and they clearly separate as two distinct peaks in the 2D IM-MS spectra of the mixture. The identification is further verified by the comparison of the CCS values recorded for the individual peaks against the reference CCS values in the Unified CCS Compendium PCDL, with a deviation of less than 0.7%. As shown in Fig. 4, high resolution demultiplexing produces narrower peaks of higher intensity and slightly clearer separation of sucrose and maltose, compared to the standard demultiplexing. The postacquisition data reconstruction technique of high resolution demultiplexing (HRdm) clearly improved resolution in the ion mobility dimension (May et al., 2020). While distinction between isobaric disaccacharides is a property of the general ion mobility capabilities of the 6560 (and enhanced by HRdm processing), it provides an additional criterion for identification confidence available for the RHIMMS method.

### Application of RHIMMS to bacterial metabolomes

**Fig. 5:**
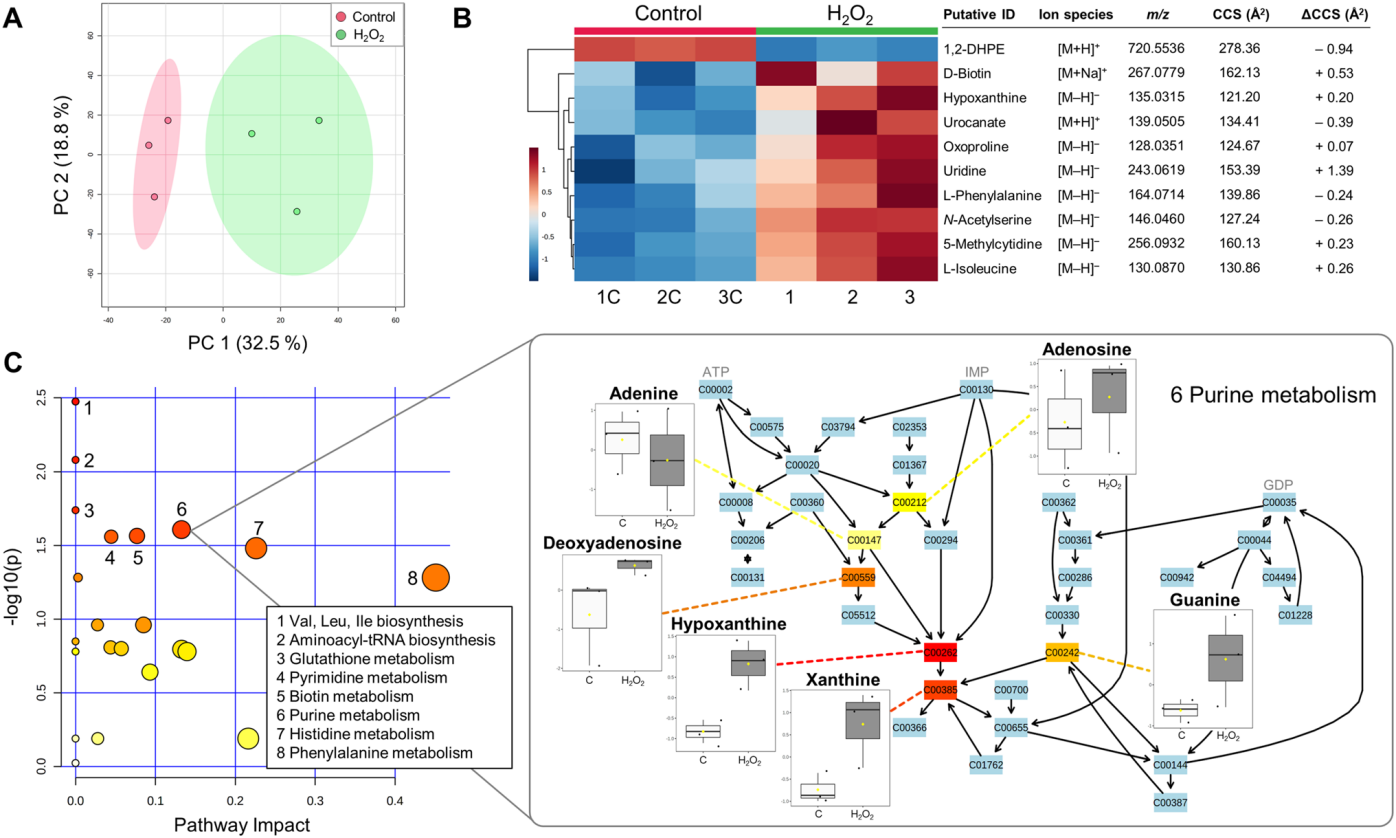
Metabolomics analysis of *Rhizobium* cells treated with 2 mM H_2_O_2_, as generated by MetaboAnalyst 5.0. **A** PCA scores plot indicating differences in metabolic profiles in H_2_O_2_-treated and control (untreated) samples (n = 3); **B** heat map depicting the top 10 most significantly affected metabolites after H_2_O_2_ treatment, annotated using the Unified CCS Compendium. The experimentally measured CCS values and ΔCCS values (measured CCS–Unified CCS Compendium) are presented; red indicates an increase and blue indicates a decrease, based on OD_600_-normalized and log-transformed intensities; **C** scatter plot of KEGG metabolic pathways in *Rhizobium* cells affected by H_2_O_2_ treatment, showing log p values from the pathway enrichment analysis (darker color indicates more significant changes within a pathway) and pathway impact values from the pathway topology analysis (the size of the node corresponds to the pathway impact score); significantly impacted pathways are annotated. KEGG purine metabolic pathway is focused upon as an example, depicting six putatively identified purine metabolites, out of which hypoxanthine and xanthine increased significantly after H_2_O_2_ treatment (color gradient from yellow to red indicates increasing significance values). The boxplots corresponding to the six metabolites represent the median ± IQR of OD_600_-normalized, log-transformed and auto-scaled intensities. *1,2-DHPE* 1,2-Diheptadecanoyl-sn-glycero-3-phosphoethanolamine, *ATP* adenosine triphosphate, *C* control, *GDP* guanosine diphosphate, *Ile* L-Isoleucine, *IMP* inosine monophosphate, *Leu* L-Leucine, *Val* L-Valine

To demonstrate the applicability of RHIMMS for the metabolomics analysis of complex biological samples, a plant growth-promoting bacterium, *Rhizobium leguminosarum* bv. *trifolii* was grown with and without the addition of 2 mM H_2_O_2_ (an oxidative stress inducer) for 6 h. Intracellular and extracellular metabolomes, extracted with a homogenous mixture of chloroform/methanol/water 1:3:1, were subjected to untargeted metabolomics analysis using RHIMMS (Fig. 5). The IMFE algorithm of Mass Profiler 10.0 software, which uses retention time, ion mobility drift time and *m/z* for feature extraction, enabled the detection of 2139 molecular features (negative and positive ionization modes combined) in the intracellular metabolome. PCA performed on all these metabolic features revealed the impact of H_2_O_2_ treatment on the *Rhizobium* metabolome, clearly separating treated from untreated cells (Fig. 5A). This clustering is in accordance with the observation that the addition of H_2_O_2_ significantly (p < 0.05) reduced the growth of the treated cells (final OD_600_ 3.8 ± 0.3) compared to untreated cells (final OD_600_ 5.1 ± 0.1) in response to oxidative stress. Compared against Unified CCS Compendium PCDL, 46 of the detected features were annotated with a higher degree of confidence, based on their *m/z* and CCS values ([M + H]^+^, [M + Na]^+^ and [M–H]^–^ ion species were taken into account; Fig. 5B).

The majority of the most significantly (p < 0.05) affected metabolites, including the possible glutathione metabolism product 5′-oxoproline or pyroglutamate (Niehaus et al., 2017), were upregulated in the treated samples.

Hydrogen peroxide is a strong oxidizing agent, disrupting iron-sulphur clusters and oxidizing sulphur-containing amino acids in proteins, thus causing cellular damage (Ezraty et al., 2017; Lemire et al., 2017). Changes in the metabolic profiles of *Rhizobium* cells as a result of H_2_O_2_ treatment were clearly observed using the RHIMMS method. The pathway enrichment analysis revealed several pathways significantly impacted by H_2_O_2_ treatment, including amino acid metabolism (phenylalanine, histidine, valine, leucine and isoleucine pathways), glutathione, biotin, pyrimidine and purine metabolic pathways (Fig. 5C). Within purine metabolism, six metabolites were detected and the relative levels of two of them—hypoxanthine and xanthine—significantly increased after the addition of H_2_O_2_ (p ≤ 0.05). Changes were also observed in the metabolic profiles of some of the 348 molecular features in the extracellular metabolome of the treated cultures. Fourteen of these features were annotated with reference to the Unified CCS Compendium, out of which hypoxanthine alone was significantly affected by the treatment (Fig. S2).

While 2% (46 of the intracellular annotations) or 4% (14 of the extracellular annotations) is a relatively small proportion of the total number of detected, annotated features, CCS values represent a valuable exclusionary criteria for isobaric false positives. In the future, as experimentally derived CCS libraries increase in size, these libraries will become a more powerful resource for increasing identification confidence. In this case, the library used—the Unified CCS Compendium (Picache et al., 2019), is restricted to 3,800 experimentally determined CCS values for metabolites. The AllCCS compendium, which contains more than two million theoretical CCS values was recently published by Zhou et al. (2020) and could be expected to substantially increase the number of annotations in this study at a cost of increased false positives, but it is not currently searchable with large datasets. Our hope is that when the Unified CCS compendium expands, or AllCCS becomes available to search fully, increased confidence in identification derived from CCS values can be obtained from the same dataset.

## Conclusions

We evaluated RHIMMS, a rapid LC-IM-MS method utilizing a short HILIC-Z column in combination with DTIM-qTOF to assess its suitability for routine metabolomics in a high throughput laboratory. Running RHIMMS both in positive and negative ionization modes enabled the annotation of a large number of molecular features and coverage of a broad range of metabolites (comprising both polar and non-polar). Good reproducibility of the method in complex matrix conditions was evidenced by the analysis of hundreds of consecutive injections of a chicken serum extract. Thousands of molecular features were extracted in the serum metabolome using this express method, with a 3.5-min acquisition time per injection. Chromatography alone compares favourably to our previous routine 30-min ZIC-pHILIC-DTIM-qTOF. Ion mobility spectrometry provided additional peak capacity and the potential to distinguish isomers based on their ion mobility drift time. An additional dimension of confidence was achieved for feature annotations CCS values in addition to chromatographic retention time and accurate mass. Untargeted metabolomics analysis of a bacterial metabolome proved RHIMMS to be a suitable method for comparative metabolomics studies in complex mixtures.

## Supplementary Information

Below is the link to the electronic supplementary material.

Supplementary file1 (DOCX 1563 KB)

## Data Availability

The metabolomics and metadata reported in this paper are available via MetaboLights: https://www.ebi.ac.uk/metabolights/editor/www.ebi.ac.uk/metabolights/MTBLS2907 with study identifier MTBLS2907.
