# Supplementary material for: Rapid HILIC-Z ion mobility mass spectrometry (RHIMMS) method for untargeted metabolomics of complex biological samples
Source: Metabolomics. 2022 Feb 28;18(3):16. doi: 10.1007/s11306-022-01871-1 (PMC8885480; doi:10.1007/s11306-022-01871-1)
Supplement: Supplementary file 1 — Supplementary file1 (DOCX 1563 KB) [file 11306_2022_1871_MOESM1_ESM.docx]

Supporting Information

Rapid HILIC-Z Ion Mobility Mass Spectrometry (RHIMMS) Method for Untargeted Metabolomics of Complex Biological Samples

Martina Pičmanová^1^, Tessa Moses^2^, Joan Cortada-Garcia^1^, Georgina Barrett^1^, Hannah Florance^3^, Sufyan Pandor^3^, Karl Burgess^1,2^*

^1^Institute of Quantitative Biology, Biochemistry and Biotechnology, University of Edinburgh, Max Born Crescent, Edinburgh EH9 3BF, United Kingdom

^2^EdinOmics, University of Edinburgh, Max Born Crescent, Edinburgh EH9 3BF, United Kingdom

^3^Agilent Technologies UK Limited, Cheadle Royal Business Park Stockport, Cheshire SK8 3GR, United Kingdom

*****Correspondence: karl.burgess@ed.ac.uk

Table of contents:

Table S1 S2

Table S2……………………………………………………………………………………………………………………………………………S3

Figure S1 S7

Figure S2 S8

| **IM-qTOF (Agilent 6560)** | | |
| --- | --- | --- |
| Ionization polarity | **Positive** | **Negative** |
| Gas temperature | 225 °C | 225 °C |
| Drying gas | 13 L/min | 13 L/min |
| Nebulizer pressure | 60 psi | 60 psi |
| Sheath gas temperature | 340 °C | 340 °C |
| Sheath gas flow | 12 L/min | 12 L/min |
| Capillary voltage | 3000 V | 3000 V |
| Nozzle voltage | 200 V | 200 V |
| Fragmentor | 395 V | 395 V |
| Octopole voltage | 750 V | 750 V |
| Acquisition range | 50-1700 m/z | 50-1700 m/z |
| MS acquisition rate | 0.8 frames/s | 0.8 frames/s |
| IM drift tube |  |  |
| Entrance voltage | 1250 V | −1175 V |
| Exit voltage | 250 V | −250 V |
| Pressure | 3.940 Torr | 3.940 Torr |
| Temperature | 31.3 °C | 31.5 °C |
| Gas | N_2_ | N_2_ |
| IM front funnel |  |  |
| High pressure funnel delta | 150 V | −120 V |
| High pressure funnel RF | 120 V | −100 V |
| Trap funnel delta | 180 V | −180 V |
| Trap funnel exit | 10 V | −10 V |
| Trap funnel RF | 180 V | −80 V |
| IM rear funnel |  |  |
| IM hex entrance | 41 V | −41 V |
| IM hex delta | −8 V | 8 V |
| IM hex RF | 600 V | −600 V |
| Rear funnel entrance | 240 V | −240 V |
| Rear funnel exit | 43 V | −43 V |
| Rear funnel RF | 130 V | −90 V |
| IM trap |  |  |
| Trap fill time | 2500 μs | 2500 μs |
| Trap release time | 250 μs | 250 μs |
| Trap entrance | 91 V | −91 V |
| Trap entrance grid delta | 10 V | −10 V |
| Trap entrance grid low | 96 V | −96 V |
| Trap exit | 90 V | −90 V |
| Trap exit grid 1 delta | 4 V | −5 V |
| Trap exit grid 1 low | 88.5 V | −86 V |
| Trap exit grid 2 delta | 8.5 V | −10.5 V |
| Trap exit grid 2 low | 87 V | −84.9 V |

**Table S1.** Optimized IM-qTOF parameters used on the Agilent 6560.

**Table S2.** Intra-batch data consistency and reproducibility based on multiple injections of chicken serum extract using RHIMMS. The average retention time (RT) and area of the top 50 most intense annotated features, along with their deviation across 200 consecutive injections (recommended batch size) in positive and negative ionization modes are tabulated.

| **Annotation** | **Formula** | **Ion Species** | **Database** | | **RT** | | **Area (x10^6^)** | |  |
| --- | --- | --- | --- | --- | --- | --- | --- | --- | --- |
|  |  |  | **CCS (Å^2^)** | **Mass (Th)** | **Average** | **%RSD** | **Average** | **%RSD** |  |
| ***Positive Ionization mode (n=200)*** | | | | | | | | | |
| LysoPC (16:0) | C_24_H_50_NO_7_P | (M+H)^+^ | 231.4 | 495.3325 | 1.23 | 0.16 | 121.34 | 9.96 |  |
| LysoPC (18:0) | C_26_H_54_NO_7_P | (M+H)^+^ | 238.8 | 523.3638 | 1.18 | 0.26 | 116.36 | 7.63 |  |
| SM 34:01 | C_39_H_79_N_2_O_6_P | (M+H)^+^ |  | 702.5676 | 1.07 | 0.28 | 113.45 | 4.31 |  |
| PC (18:1/18:1) (del9-trans) | C_44_H_84_NO_8_P | (M+H)^+^ | 293.3 | 785.5935 | 0.60 | 0.50 | 79.70 | 4.10 |  |
| PC 36:01 | C_44_H_86_NO_8_P | (M+H)^+^ |  | 787.6091 | 0.62 | 0.65 | 51.72 | 7.25 |  |
| PC (18:1(9Z)/16:0) | C_42_H_82_NO_8_P | (M+H)^+^ | 287.9 | 759.5778 | 0.62 | 5.83 | 58.13 | 12.12 |  |
| Betaine | C_5_H_11_NO_2_ | (M+H)^+^ | 121.1 | 117.079 | 1.48 | 0.20 | 32.98 | 1.90 |  |
| PC 38:04 | C_46_H_84_NO_8_P | (M+H)^+^ |  | 809.5935 | 0.56 | 0.72 | 28.65 | 5.39 |  |
| LysoPC (18:1) | C_26_H_52_NO_7_P | (M+H)^+^ | 233.2 | 521.3481 | 1.18 | 1.70 | 21.74 | 8.11 |  |
| PC 34:00 | C_42_H_84_NO_8_P | (M+H)^+^ |  | 761.5935 | 0.71 | 2.41 | 18.46 | 17.81 |  |
| PE 37:02 | C_42_H_80_NO_8_P | (M+H)^+^ |  | 757.5622 | 0.61 | 0.49 | 23.76 | 4.74 |  |
| PC 38:03 | C_46_H_86_NO_8_P | (M+H)^+^ |  | 811.6091 | 0.57 | 4.77 | 18.21 | 8.83 |  |
| PS 36:01 | C_42_H_80_NO_10_P | (M+H)^+^ | 285.6 | 789.552 | 0.73 | 2.74 | 9.63 | 11.06 |  |
| 1,2-Dipalmitoyl-sn-glycero-3-phosphocholine | C_40_H_80_NO_8_P | (M+H)^+^ | 284.6 | 733.5622 | 0.67 | 2.24 | 8.61 | 25.62 |  |
| PC 36:02 HETE | C_44_H_84_NO_9_P | (M+H)^+^ |  | 801.5884 | 0.67 | 5.24 | 11.69 | 12.16 |  |
| PE 37:03 | C_42_H_78_NO_8_P | (M+H)^+^ |  | 755.5465 | 0.68 | 8.44 | 6.69 | 18.11 |  |
| PS 38:02 | C_44_H_82_NO_10_P | (M+H)^+^ |  | 815.5676 | 0.70 | 2.73 | 7.81 | 26.85 |  |
| L-Phenylalanine | C_9_H_11_NO_2_ | (M+H)^+^ |  | 165.079 | 2.40 | 0.63 | 5.09 | 24.76 |  |
| PC 36:01 HETE | C_44_H_86_NO_9_P | (M+Na)^+^ |  | 803.604 | 0.64 | 2.82 | 4.74 | 21.10 |  |
| PS 38:01 | C_44_H_84_NO_10_P | (M+H)^+^ |  | 817.5833 | 0.67 | 18.80 | 17.57 | 18.79 |  |
| 1,2-Distearoyl-sn-glycero-3-phosphocholine | C_44_H_88_NO_8_P | (M+H)^+^ | 297.6 | 789.6248 | 0.87 | 6.00 | 4.03 | 17.59 |  |
| PS 40:03 | C_46_H_84_NO_10_P | (M+H)^+^ |  | 841.5833 | 0.71 | 3.97 | 9.77 | 2.52 |  |
| Platelet-activating Factor | C_26_H_54_NO_7_P | (M+Na)^+^ |  | 523.3638 | 1.16 | 0.17 | 2.84 | 10.10 |  |
| PE 36:02 | C_41_H_78_NO_8_P | (M+H)^+^ |  | 743.5465 | 0.74 | 0.68 | 2.28 | 3.07 |  |
| SM 40:01 | C_45_H_91_N_2_O_6_P | (M+H)^+^ | 299.1 | 786.6615 | 1.29 | 0.54 | 2.54 | 32.19 |  |
| Neomycin B | C_23_H_46_N_6_O_13_ | (M+Na)^+^ |  | 614.3123 | 0.21 | 0.47 | 2.31 | 6.84 |  |
| SM 38:01 | C_43_H_87_N_2_O_6_P | (M+H)^+^ | 293.4 | 758.6302 | 1.11 | 0.81 | 2.16 | 4.74 |  |
| Hypoxanthine | C_5_H_4_N_4_O | (M+H)^+^ |  | 136.0385 | 0.74 | 0.27 | 2.04 | 3.09 |  |
| PC (16:0E/2:0) | C_26_H_54_NO_7_P | (M+Na)^+^ | 240.6 | 523.3638 | 1.16 | 0.17 | 2.00 | 7.78 |  |
| Roxithromycin | C_41_H_76_N_2_O_15_ | (M+NH_4_)^+^ |  | 836.5246 | 0.81 | 0.75 | 2.95 | 23.20 |  |
| PC 32:01 | C_40_H_78_NO_8_P | (M+H)^+^ | 277.6 | 731.5465 | 0.63 | 0.63 | 1.90 | 20.27 |  |
| LysoPC (13:0) | C_21_H_44_NO_7_P | (M+H)^+^ |  | 453.2855 | 1.36 | 0.22 | 1.82 | 9.28 |  |
| L-Arginine | C_6_H_14_N_4_O_2_ | (M+H)^+^ |  | 174.1117 | 2.71 | 0.15 | 1.87 | 13.54 |  |
| (S)-Taurocholic Acid | C_26_H_45_NO_7_S | (M+H)^+^ |  | 515.2917 | 0.30 | 0.66 | 1.76 | 2.08 |  |
| PC 38:02 | C_46_H_88_NO_8_P | (M+H)^+^ |  | 813.6248 | 0.37 | 2.71 | 2.04 | 7.96 |  |
| SM 42:01 | C_47_H_95_N_2_O_6_P | (M+H)^+^ | 304.4 | 814.6928 | 1.59 | 0.56 | 1.78 | 11.41 |  |
| LysoPC (P-18:0) | C_26_H_54_NO_6_P | (M+H)^+^ | 235.5 | 507.3689 | 1.23 | 0.65 | 1.90 | 9.77 |  |
| PC 40:06 | C_48_H_84_NO_8_P | (M+H)^+^ |  | 833.5935 | 0.55 | 2.38 | 1.78 | 8.93 |  |
| PC 40:05 | C_48_H_86_NO_8_P | (M+H)^+^ |  | 835.6091 | 0.55 | 0.72 | 1.91 | 4.37 |  |
| SM 36:02 | C_41_H_81_N_2_O_6_P | (M+H)^+^ | 285.3 | 728.5832 | 1.03 | 0.19 | 1.53 | 2.88 |  |
| SM 41:01 | C_46_H_93_N_2_O_6_P | (M+H)^+^ | 302.3 | 800.6771 | 1.32 | 0.83 | 1.13 | 24.51 |  |
| PC 40:04 | C_48_H_88_NO_8_P | (M+H)^+^ |  | 837.6248 | 0.56 | 3.06 | 1.32 | 11.70 |  |
| TGQIK (Tryptic Peptide) | C_23_H_44_N_7_O_8_ | (M+NH_4_)^+^ |  | 546.3251 | 0.30 | 0.66 | 1.10 | 2.53 |  |
| SM 40:02 | C_45_H_89_N_2_O_6_P | (M+H)^+^ | 296.8 | 784.6458 | 1.03 | 0.39 | 1.12 | 4.22 |  |
| PE 38:04 | C_43_H_78_NO_8_P | (M+H)^+^ |  | 767.5465 | 0.69 | 0.58 | 1.17 | 25.52 |  |
| PE 35:01 | C_40_H_78_NO_8_P | (M+H)^+^ |  | 731.5465 | 0.37 | 3.26 | 1.07 | 14.51 |  |
| PC 36:00 HETE | C_44_H_88_NO_9_P | (M+Na)^+^ |  | 805.6197 | 0.62 | 11.38 | 2.29 | 17.14 |  |
| PC (16:0/2:0) | C_26_H_52_NO_8_P | (M+Na)^+^ |  | 537.3431 | 1.31 | 0.46 | 1.07 | 5.39 |  |
| LysoPC (19:0) | C_27_H_56_NO_7_P | (M+H)^+^ | 240.5 | 537.3794 | 1.16 | 0.17 | 1.01 | 10.67 |  |
| PS 40:02 | C_46_H_86_NO_10_P | (M+H)^+^ |  | 843.5989 | 0.68 | 6.61 | 4.27 | 8.05 |  |
| ***Negative ionization mode (n=200)*** | | | | | | | | | |
| Oleic Acid (18:1) | C_18_H_34_O_2_ | (M-H)^-^ | 176.2 | 282.2559 | 0.28 | 1.08 | 11.05 | 3.52 |  |
| Linoleic Acid (18:2) | C_18_H_32_O_2_ | (M-H)^-^ | 174.6 | 280.2402 | 0.28 | 1.43 | 6.89 | 5.27 |  |
| Palmitate | C_16_H_32_O_2_ | (M-H)^-^ | 168.7 | 256.2402 | 0.31 | 3.91 | 6.73 | 29.01 |  |
| Stearic Acid (18:0) | C_18_H_36_O_2_ | (M-H)^-^ | 177.3 | 284.2715 | 0.28 | 1.07 | 5.38 | 4.70 |  |
| L-(+)-Lactic Acid | C_3_H_6_O_3_ | (M-H)^-^ |  | 90.0317 | 1.48 | 0.47 | 4.88 | 11.92 |  |
| Trifluoroacetic Acid | C_2_HF_3_O_2_ | (M-H)^-^ |  | 113.9929 | 1.50 | 1.13 | 3.51 | 30.40 |  |
| Arachidonic Acid (20:4) | C_20_H_32_O_2_ | (M-H)^-^ | 181.6 | 304.2402 | 0.27 | 1.48 | 2.13 | 11.39 |  |
| Isocitrate | C_6_H_8_O_7_ | (M-H)^-^ | 127.3 | 192.027 | 2.72 | 0.40 | 1.39 | 28.35 |  |
| Xanthine | C_5_H_4_N_4_O_2_ | (M-H)^-^ | 122.2 | 152.0334 | 0.74 | 0.41 | 1.32 | 6.77 |  |
| Oxoproline | C_5_H_7_NO_3_ | (M-H)^-^ | 124.6 | 129.0426 | 1.82 | 0.16 | 1.31 | 3.25 |  |
| 9-HODE | C_18_H_32_O_3_ | (M-H)^-^ | 180 | 296.2351 | 0.35 | 0.85 | 1.14 | 7.99 |  |
| Palmitoleic Acid (16:1) | C_16_H_30_O_2_ | (M-H)^-^ | 167.8 | 254.2246 | 0.31 | 4.56 | 1.21 | 29.29 |  |
| L-Phenylalanine | C_9_H_11_NO_2_ | (M-H)^-^ |  | 165.079 | 1.63 | 0.24 | 0.86 | 7.59 |  |
| 8(S)-HETE | C_20_H_32_O_3_ | (M-H)^-^ |  | 320.2351 | 0.35 | 0.86 | 0.84 | 16.79 |  |
| Bishomo-gamma-linolenic Acid (20:3) | C_20_H_34_O_2_ | (M-H)^-^ | 182 | 306.2559 | 0.27 | 1.11 | 0.74 | 7.21 |  |
| PS 40:01 | C_46_H_88_NO_10_P | (M-H)^-^ |  | 845.6146 | 0.62 | 0.97 | 0.53 | 15.68 |  |
| Pyroglutamate | C_5_H_7_NO_3_ | (M-H)^-^ |  | 129.0426 | 1.82 | 0.17 | 0.57 | 4.69 |  |
| Uridine | C_9_H_12_N_2_O_6_ | (M-H)^-^ | 152 | 244.0695 | 0.59 | 0.17 | 0.44 | 8.56 |  |
| trans-4-Hydroxy-L-proline | C_5_H_9_NO_3_ | (M-H)^-^ |  | 131.0582 | 2.37 | 0.21 | 0.45 | 9.95 |  |
| Sorbose | C_6_H_12_O_6_ | (M-H)^-^ |  | 180.0634 | 2.35 | 0.17 | 0.47 | 12.74 |  |
| L-Isoleucine | C_6_H_13_NO_2_ | (M-H)^-^ | 130.6 | 131.0946 | 1.67 | 0.42 | 0.37 | 5.77 |  |
| Taurine | C_2_H_7_NO_3_S | (M-H)^-^ | 118.5 | 125.0147 | 1.99 | 0.15 | 0.33 | 4.75 |  |
| 12(R)-HETE | C_20_H_32_O_3_ | (M-H)^-^ | 183.5 | 320.2351 | 0.25 | 4.84 | 0.35 | 16.41 |  |
| L-Tyrosine | C_9_H_11_NO_3_ | (M-H)^-^ | 144.5 | 181.0739 | 2.06 | 0.24 | 0.31 | 4.26 |  |
| (S)-Taurodeoxycholic Acid | C_26_H_45_NO_6_S | (M-H)^-^ |  | 499.2968 | 0.59 | 2.38 | 0.29 | 9.62 |  |
| Glutamine | C_5_H_10_N_2_O_3_ | (M-H)^-^ | 127.7 | 146.0691 | 2.60 | 0.08 | 0.47 | 6.80 |  |
| PS 38:01 | C_44_H_84_NO_10_P | (M-H)^-^ |  | 817.5833 | 0.63 | 0.95 | 0.28 | 11.33 |  |
| Hypoxanthine | C_5_H_4_N_4_O | (M-H)^-^ | 121 | 136.0385 | 0.67 | 0.15 | 0.28 | 9.49 |  |
| L-(+)-Tartartic Acid | C_4_H_6_O_6_ | (M-H)^-^ |  | 150.0164 | 0.22 | 0.46 | 0.25 | 14.80 |  |
| Carnosine | C_9_H_14_N_4_O_3_ | (M-H)^-^ | 153.1 | 226.1066 | 2.87 | 0.21 | 0.23 | 45.29 |  |
| PE 36:02 | C_41_H_78_NO_8_P | (M-H)^-^ | 272 | 743.5465 | 0.75 | 0.80 | 0.23 | 38.59 |  |
| LysoPC (13:0) | C_21_H_44_NO_7_P | (M-H)^-^ |  | 453.2855 | 1.37 | 0.29 | 0.21 | 6.02 |  |
| 9-OxoODE | C_18_H_30_O_3_ | (M-H)^-^ |  | 294.2195 | 0.34 | 0.58 | 0.23 | 15.34 |  |
| Thymidine | C_10_H_14_N_2_O_5_ | (M-H)^-^ | 154.2 | 242.0903 | 0.39 | 0.26 | 0.18 | 19.70 |  |
| Leukotriene B4 | C_20_H_32_O_4_ | (M-H)^-^ | 186.8 | 336.2301 | 0.54 | 7.08 | 0.21 | 24.25 |  |
| Petroselinate | C_18_H_34_O_2_ | (M-H)^-^ |  | 282.2559 | 0.28 | 2.17 | 0.22 | 19.66 |  |
| Anserine | C_10_H_16_N_4_O_3_ | (M-H)^-^ | 154.9 | 240.1222 | 2.79 | 0.14 | 0.16 | 29.38 |  |
| Purine | C_5_H_4_N_4_ | (M-H)^-^ | 114.6 | 120.0436 | 0.62 | 8.01 | 0.61 | 30.46 |  |
| N,N-Dimethylglycine Ethyl Ester | C_6_H_13_NO_2_ | (M-H)^-^ |  | 131.0946 | 1.67 | 0.42 | 0.22 | 14.95 |  |
| 5(S)-HETE | C_20_H_32_O_3_ | (M-H)^-^ | 186.6 | 320.2351 | 0.38 | 14.06 | 0.25 | 44.85 |  |
| PE 38:04 | C_43_H_78_NO_8_P | (M-H)^-^ | 275.9 | 767.5465 | 0.69 | 0.87 | 0.15 | 39.01 |  |
| alpha-Linolenic acid (PC18:3) | C_18_H_30_O_2_ | (M-H)^-^ | 173.9 | 278.2246 | 0.28 | 8.21 | 0.21 | 33.21 |  |
| N-alpha-Acetyllysine | C_8_H_16_N_2_O_3_ | (M-H)^-^ |  | 188.1161 | 2.43 | 0.21 | 0.14 | 8.75 |  |
| Myoinositol | C_6_H_12_O_6_ | (M-H)^-^ | 131.5 | 180.0634 | 2.35 | 0.17 | 0.22 | 18.88 |  |
| Citramalic Acid | C_5_H_8_O_5_ | (M-H)^-^ | 123.1 | 148.0372 | 2.73 | 0.40 | 0.17 | 9.05 |  |
| PS 42:03 | C_48_H_88_NO_10_P | (M-H)^-^ |  | 869.6146 | 0.58 | 1.03 | 0.12 | 19.83 |  |
| Margaric Acid | C_17_H_34_O_2_ | (M-H)^-^ | 173.1 | 270.2559 | 0.30 | 4.71 | 0.16 | 29.50 |  |
| D-(+)-Malate | C_4_H_6_O_5_ | (M-H)^-^ | 116.3 | 134.0215 | 2.74 | 0.29 | 0.17 | 9.30 |  |
| Lauric Acid (12:0) | C_12_H_24_O_2_ | (M-H)^-^ | 154.2 | 200.1776 | 0.33 | 1.85 | 0.14 | 12.34 |  |
| Eicosadienoic Acid (20:2) | C_20_H_36_O_2_ | (M-H)^-^ | 181.8 | 308.2715 | 0.27 | 1.47 | 0.12 | 8.67 |  |

**Figure S1**. Inter-batch effect observed for RHIMMS. Two batches of chicken serum extract (100 injections each) in negative and positive ionisation modes, were compared using PCA score plots. The two serum batches form two separated clusters, based on 3,604 and 7,317 molecular features for negative and positive ionisation modes, respectively.


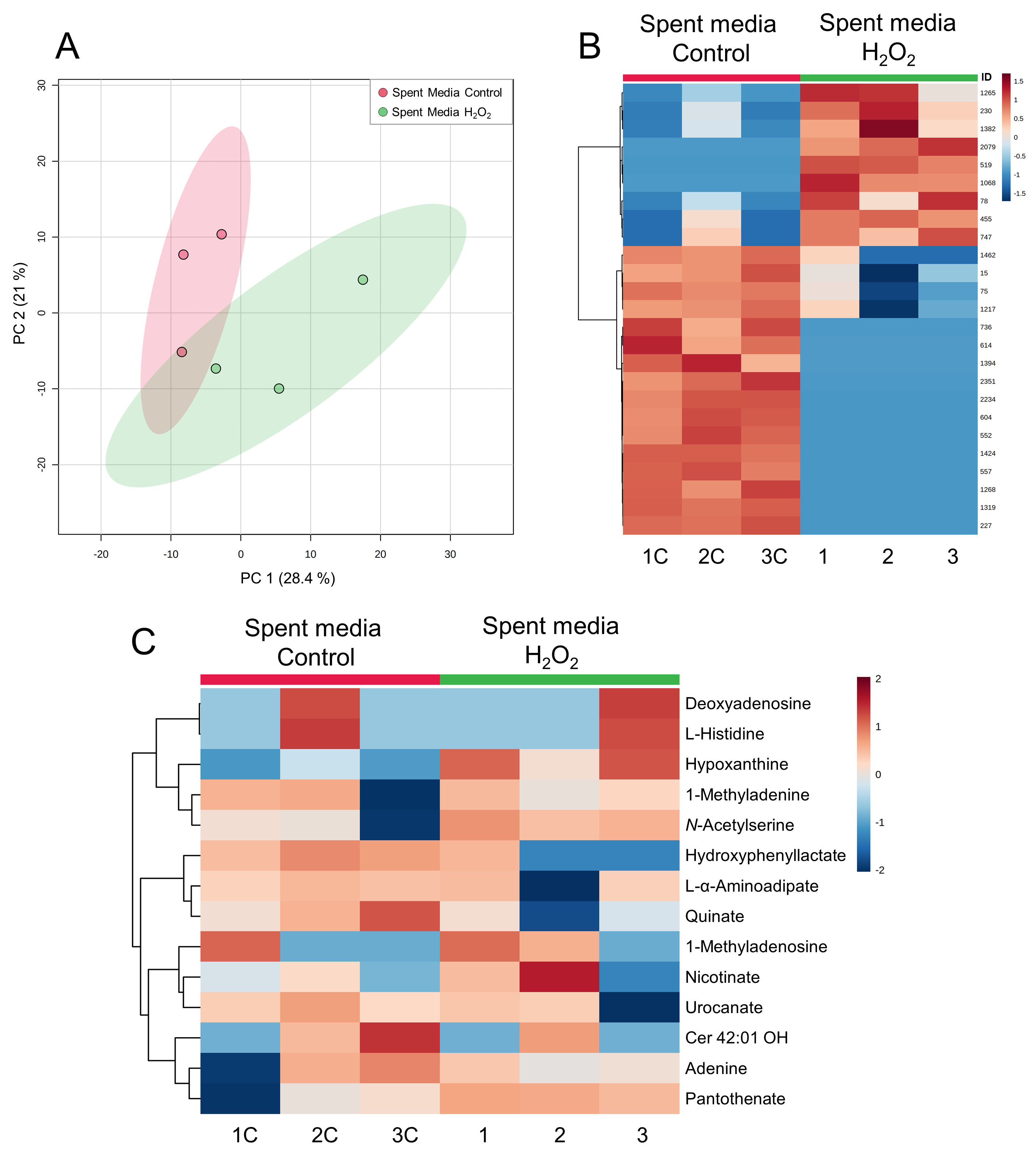


Figure S2. Metabolomics analysis of spent media from *Rhizobium* cultures treated with 2 mM H_2_O_2_, as generated by MetaboAnalyst 5.0. (A) PCA scores plot indicating differences in metabolic profiles in H_2_O_2_-treated and control samples (n = 3), based on 348 extracted molecular features; (B) Heat map depicting the top 25 most significantly affected molecular features; (C) Heat map showing all metabolites in spent media after H_2_O_2_ treatment which were annotated using McLean CCS Compendium PCDL (Picache *et al.*, 2019). None of them was significantly affected by H_2_O_2_, with the exception of hypoxanthine. All molecular features present in the fresh media, which were co-incubated together with *Rhizobium* cultures, were removed as the background from the dataset.
